# Population dynamics and socio-spatial organization of the Aurignacian: Scalable quantitative demographic data for western and central Europe

**DOI:** 10.1371/journal.pone.0211562

**Published:** 2019-02-13

**Authors:** Isabell Schmidt, Andreas Zimmermann

**Affiliations:** Institute of Prehistoric Archaeology, CRC806, University of Cologne, Cologne, Germany; University at Buffalo - The State University of New York, UNITED STATES

## Abstract

Demographic estimates are presented for the Aurignacian techno-complex (~42,000 to 33,000 y calBP) and discussed in the context of socio-spatial organization of hunter-gatherer populations. Results of the analytical approach applied estimate a mean of 1,500 persons (upper limit: 3,300; lower limit: 800) for western and central Europe. The temporal and spatial analysis indicates an increase of the population during the Aurignacian as well as marked regional differences in population size and density. Demographic increase and patterns of socio-spatial organization continue during the subsequent early Gravettian period. We introduce the concept of Core Areas and Extended Areas as informed analytical spatial scales, which are evaluated against additional chronological and archaeological data. Lithic raw material transport and personal ornaments serve as correlates for human mobility and connectedness in the interpretative framework of this study. Observed regional differences are set in relation with the new demographic data. Our large-scale approach on Aurignacian population dynamics in Europe suggests that past socio-spatial organization followed socially inherent rules to establish and maintain a functioning social network of extremely low population densities. The data suggest that the network was fully established across Europe during the early phase of the Gravettian, when demographic as well as cultural developments peaked.

## Introduction

A key issue in Paleolithic research is the understanding of how demographic, social, technological and environmental factors influenced the successful spread and establishment of anatomically modern Humans across Europe. It is commonly accepted that the Aurignacian techno-complex constitutes a pan-European phenomenon [1, 2]. Its homogeneous archaeological appearance at this scale has favored models considering fast-spreading, highly mobile and interconnected populations. Yet, explanatory models of biological and cultural developments compete on mode and pace. This is commonly due to the poor chronological resolution, limited anthropological evidence, and the ongoing discussion on the internal and regionally heterogeneous chrono-cultural structure of the paleoanthropological and archaeological record itself [3–7].

Studies explicitly concerned with Early Upper Paleolithic demography are scarce, although explicit or implicit references to this field are common in the literature. Currently available estimates operate only at single spatial scales, using either regional [8, 9] or pan-European [10–12] frameworks to deduce relative frequencies or densities. Since we expect a highly uneven distribution of hunter-gatherer populations across the European landscape [13], these results hardly allow for up- and downscaling, and thus hinder comparisons with additional archaeological or related contextual data. Large-scale studies on population dynamics are generally dominated by environmentally determined approaches; in contrast, an abundance of social concepts are applied in studies at smaller, regional or local, scales.

To fill this lack of quantitative demographic data and to provide scalable results, our study presents estimates on regional and pan-European population sizes and densities for the Aurignacian techno-complex. Also providing a framework to bridge these scales, this article discusses the structuring power of human social organization for large-scale explanatory models on Upper Paleolithic societies. This neither intends to deny the impact of environmental factors on hunter-gatherers ([13], for recent advances see: [14, 15]), nor to superimpose a social architecture in a „top-down approach”([1]:34). Instead, by introducing site density-dependent derived population estimates at different temporal and spatial scales into model building processes, we argue that new information and hypotheses on large-scale socio-spatial organization can be derived. We use a newly developed and consistently tested approach [16–19] to estimate hunter-gatherer population sizes and densities for the Aurignacian techno-complex in Europe. This approach allows integration of additional data at appropriate spatial scales. Synchronically, we compare the results against archaeological proxies for mobility and interconnectedness, and diachronically follow developments during the Aurignacian and towards the Gravettian. A large-scale approach certainly involves simplification of current archaeological knowledge and ongoing controversies; however, “sometimes gross simplification can expose regularities that continual attention to complexities would hide” [20].

## Materials and methods

The defined period under investigation covers the Aurignacian techno-complex in Europe, spanning roughly from 42–33 ky calBP [21]. Paleoclimatically, it comprises the Greenland Interstadials 11 to 6 and Heinrich Event 4, the latter dated to 39.8–37.9 ky calBP [22]. The beginning of the Aurignacian is defined by the onset of the Proto and Early Aurignacian. Assemblages attributed to so-called “transitional” industries of probably Neanderthal or yet unknown origin were not considered in our study, since most of the well dated sequences indicate little or no overlap with the period under study [23, 24]. The end of the Aurignacian and beginning of the Gravettian seem to be regionally differentiated with older dated occurrences found in central and eastern Europe [25–28]. Contested assemblages in southern Iberia have been reclassified as an early Gravettian, based on technological investigations and critical reassessment of the collections [29]. For the purpose at hand, the cultural attribution was weighted more than the overlapping early radiocarbon dates of a few Gravettian sites (Trencianske Bohuslavice-Pod Tureckom, Henrykow 15, Dolni Vestonice IIa, Ranis) which were therefore excluded.

**Fig 1:**
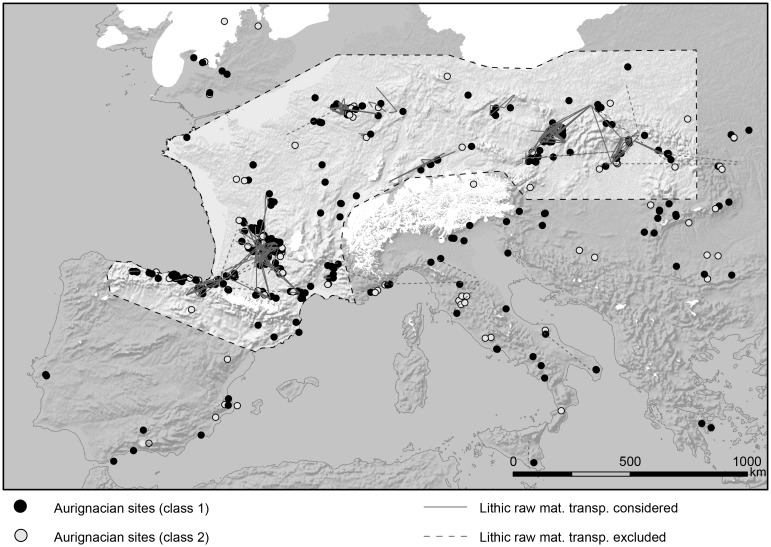
Mapping of database on assemblages / sites attributed to the Aurignacian in Europe (black: class 1, grey: class 2, i.e. excluded from calculations) and of database on raw material transport (grey lines; S1 Table). The highlighted area outlines the Total Area of Calculation (TAC, 1.5 million km^2^). Glaciers inserted from LGM-reconstruction [42] and paleocoastline at 80 m below present sea level [43].

The archaeological database [30] was compiled using information available in the literature and comprises 488 sites (Fig 1). The sites were divided based on the following criteria: The first group (class 1, n = 382) includes sites with accepted attribution based on radiometric data, stratigraphic context or diagnostic stone tools. The second group (class 2, n = 106) comprises sites for which the attribution has been critically contested in the literature, i.e. assemblages that are poorly defined, only partially or not published, or that derive from undated or undocumented contexts. Class 1 was then split into two main chronological phases: one comprising those classified as Proto or Early Aurignacian (n = 117), and the other including assemblages assigned to subsequent phases of the Aurignacian (n = 317). For the final population estimates, a critical evaluation of biases affecting the archaeological record at supraregional scales was conducted, mainly considering research intensity, preservation conditions, and visibility of the archaeological remains (see S1 File). Areas with obvious large-scale research biases, such as under-represented records or attributions still under discussion (e.g. parts of the Balkan region and Italian Peninsula, [31–35]) were excluded. We accepted well studied regions, although some areas might suffer from visibility or preservation biases, e.g. parts of northern France. Finally, southern Iberia and the British Isles are considered during discussion, but were excluded from the geostatistical analysis since occupation is confined to the final phase of the Aurignacian [29, 36, 37] and therefore expected to produce a distinct site-density pattern.

Our literature-based survey of lithic raw material transport data compiled 409 source-to-site distances as-the-crow-flies, deriving from 92 assemblages from 87 sites (see S1 Table). ‘Local’ procurement of raw material was generally considered by creating a 5 km radius buffer around each site [38], while recording of non-local, long-distance raw material transport focused on evidence from economically relevant quantities, excluding transported single pieces from our calculation.

The density-based method applied in this study has already been comprehensively described and discussed [16–19, 39–41], and applied to a series of European Upper Paleolithic contexts, such as the Gravettian [19], Last Glacial Maximum [18] and Magdalenian [16]. The approach analyses the density of archaeological sites by interpolating (Kriging) the Largest Empty Circle (LEC) distance-values obtained at each Thiessen polygon node [41]. Successive isolines are then calculated from the interpolated areal data. The “Optimal Isoline” (OI) is identified by a plateau or peak of areal increase in relation to the LEC radius, and should encircle 70–90% of the archaeological sites [41]. The OI is described by the LEC-radius value, being half of the maximum distance between two sites within the OI. In our model on scale levels (see Table 1, and S1 Fig), the OI describes the so-called Core Areas (CA, scale III), providing a geostatistically derived and site density-based measure to distinguish intensively and probably continuously occupied areas from those that were either sporadically, marginally, or not occupied. The OI in this study was identified at a radius of 29 km (S2 Fig). This means that the nearest sites located within the defined CAs are at maximum 58 km apart from each other.

**Table 1:** Overview on scale levels as well as related terms and interpretations used in the present approach. See additional explanations in S1 Fig.

Approximate Scale level		Object	Calculation value	Result at scale level	Intra- / Intermethod comparable data	Interpretation within this approach
V global	**TAC** = Total Area of Calculation	Investigated area	km^2^ of TAC	Total Population Density	Density estimate / Density estimate	Population density of areas with and without evidence of human occupation
IV supraregional	**EA** = Extended Area	Optimal Isoline and Raw Material Polygons	km^2^ of EA	Extended Area Population Density	Absolute number estimate / Density estimate	Population density of interconnected social and economic areas
III regional	**CA** = Core Area	Optimal Isoline	km^2^ of Optimal Isoline	Core Area Population Number / Density	Absolute number estimate / Density estimate	Population size and Population density of core areas of hunter-gatherer occupation
II catchment	**CatA** = Catchment Area	Raw Material Polygons	Quartile 1, 2, and 3 of km^2^ of raw material polygons	Range of catchment area size	Area size	(Minimum) size of the seasonal or annual catchment area
I local	Archaeological Sites	Archaeological techno-complex	Site coordinates	Site locale	-	Presence of hunter gatherers during a selected period

To calculate a lithic raw-material catchment area (CatA, scale II, see Table 1) for each available assemblage-based dataset, convex hulls encircling the respective spatially documented source-to-site distances were created and the area of each polygon was recorded. CatA smaller than 500 km^2^ are excluded from further analysis, as these few catchments showed unusual economic patterns of non-local raw material source use, mostly related to the specific setting of the site. Additionally, since our protocol argues that CatA represent (minimum) ranges of annually aggregating groups (see below), we do expect CatA to exceed a radius of 13 km, which corresponds to an area of approximately 500 km^2^.

The contour of the supraregional Extended Area (EA, scale IV) is formed by superimposing CatA and the CA and again creating convex hulls. Whether the EA is larger than the original CA or even unites several CAs depends on the evidence for raw material transport. Finally, the Total Area of Calculation (TAC, scale V) encompasses all areas—with or without evidence of human activity—after excluding biased (S1 File) or uninhabitable regions. The TAC defined for the present study comprises an area of around 1.5 million km^2^ (Fig 1) and encloses a total of 304 (class 1) archaeological sites, which makes up 80% of all class 1 sites.

To estimate the population size, the area of each CA is divided by the 1^st^, 2^nd^ (mean) and 3^rd^ quartile of the respective CatA (S1 Table), since we do not expect that more than one Aurignacian group exploited the same area ([13]:161 ff.). For regions with very little or no information on raw material transport, CatA data from adjacent areas has to be transferred. The results are the minimum, maximum and mean number of possible groups living in the area at the same time. To derive the absolute number of people from the estimated number of groups, we use ethno-historic data on group size. We consider them more reliable than ethno-historic data on population density. To obtain the population size, the calculated number of groups (min, max, mean) has to be multiplied by the group size, which, in the current protocol, comprises 42.5 persons. This number is the mean of people aggregated at the largest residential seasonal camp (GROUP 2, [44]) based on 16 ethno-historic hunter-gatherer groups, selected by techno-economic and environmental criteria (for details see [16]). The mean number is, however, also similar to numbers obtained from much larger samples in other studies on hunter-gatherer populations (e.g. [45] and references therein), which lends robustness to this value. Having now estimated the number of people for each CA, we use this estimate to obtain all other densities at larger spatial scales. The densities for CAs are calculated by dividing the number of people by the size (km^2^) of the CA.

Calculating population size and density for the EA scale is deemed reasonable only for areas with comprehensive information on lithic raw material transport, transferring data is not applicable. Although the available record is expected to be improved by future research, for the present study several areas are already considered to provide sufficient data, i.e. southwestern France, eastern Central European and Belgium (S1 Table). Population density for EAs is calculated dividing the number of people estimated for CA by the size of the EA. Given the larger area considered, lower population density estimates than for the CA are derived. Since reliable results are restricted to well-studied regions, EA estimates are not suitable for large scale or diachronic comparisons of densities. For those instances, we use densities estimates at the scale of the TAC: the CA population size is then divided by the size of the TAC.

## Results

**Fig 2:**
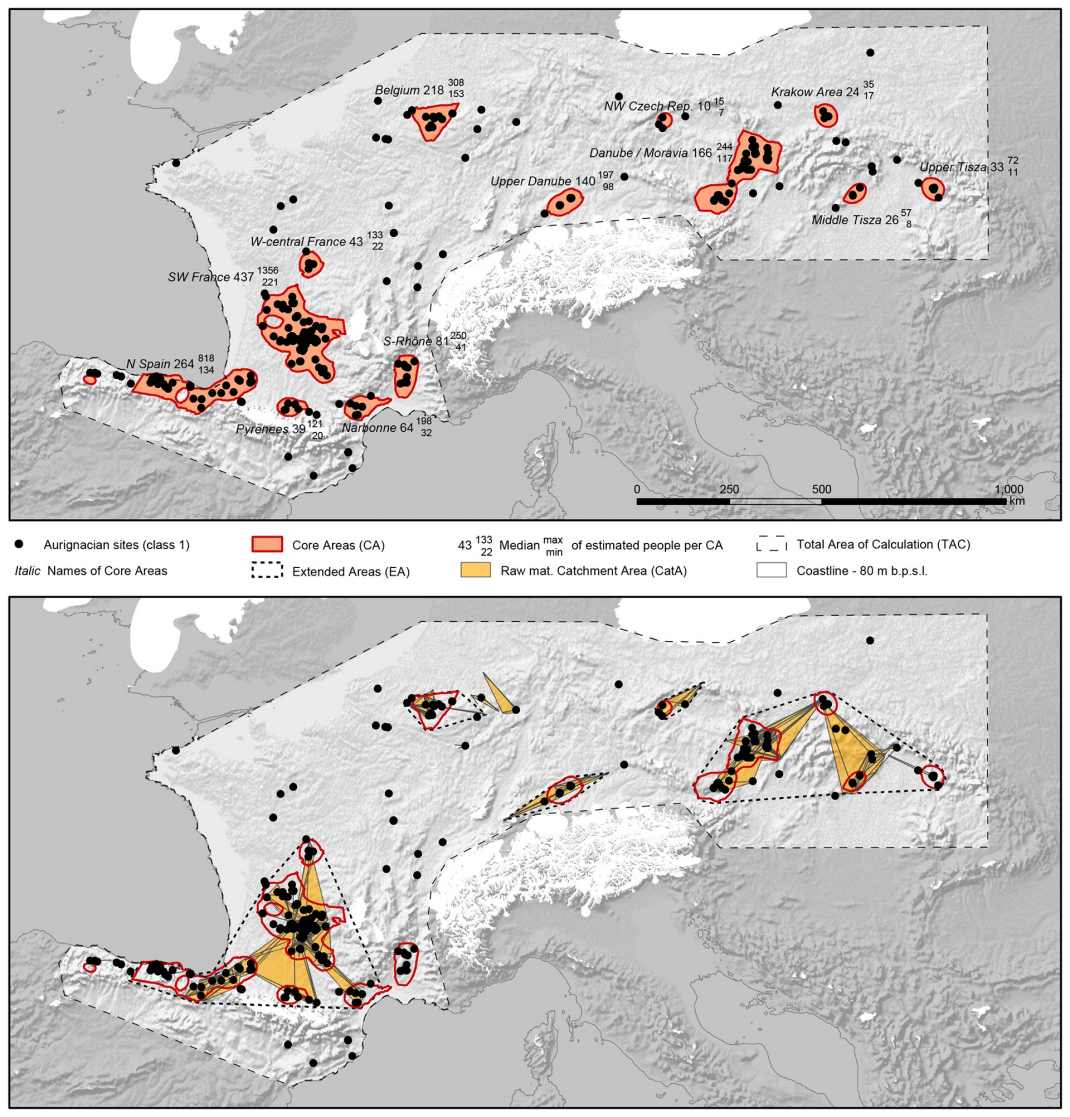
Mapping of the demographic estimates for the Aurignacian (sites: class 1) within the TAC. Upper image: Core Areas (red lines) are shown together with population density estimates, including ranges (Table 3). Lower image: Extended Areas (dashed lines) with data on raw material: solid lines connect potential source-areas of lithic raw material to sites, yellow areas indicate raw material catchments considered during the calculation of the population density estimates (S1 Table).

For the Aurignacian of western and central Europe, we estimated a population of 1,500 people, ranging from 900 to 3,800 people (Table 2). The population density estimate (given in people per 100 km^2^) is 0.103 P/100 km^2^ for the TAC (1,500,000 km^2^). 81% of all sites used in our density based approach (245 out of 304) are located within the CAs. In the present study, 13 individual CAs are identified (Table 3, Fig 2: upper). Highest estimated population numbers for the CAs are found in SW France (440 persons), N Spain (260 persons), Belgium (210), the middle Danube/Moravian (170 persons), and the upper Danube area (140 persons). Mean estimates for the remaining CAs range between 10 and 80 persons each (overall mean = 40). This interesting and marked separation of the size of the population estimates into larger (≳140) and smaller ones (<80) will be discussed later in more detail. Within the CAs the overall estimated population density centers around 1.442 P/100 km^2^. The highest densities are estimated for the CA of Belgium (mean = 3.000 P/100 km^2^), while the lowest density is reported from the Middle Danube and Krakow area (0.844).

**Table 2:** Population numbers and density estimates for all three spatial scales. Since larger scales consider areas with no or only sporadical occupation, density reduces considerably. Maximum (1), mean (2), and minimum (3) estimate of persons and population density (persons per 100km^2^).

Scale	Area (km^2^)		Number of persons	Population density
Core Areas	107,188	(1)	3805	3.549
		(2)	1545	1.442
		(3)	881	0.822
Extended Areas	302,126	(1)	3555	1.177
		(2)	1465	0.485
		(3)	840	0.278
TAC	1,500,000	(1)	3805	0.254
		(2)	1545	0.103
		(3)	881	0.059

**Table 3:** Population numbers and densities calculated for the Aurignacian Core Areas. The Core Area Population Density is given in persons per 100 km^2^. The Quartiles (Q) 1,2,3 of the size of Catchment Areas (CatA, S1 Table) and the number of datasets are listed. Only catchments located inside the TAC were considered.

Core Area	(km^2^)	Q	CatA (in km^2^)	N of CatA	Number of Groups	Number of Persons	Core Area Population Density
N Spain *	18,973	Q1	985		19.3	818	4.314
	18,973	Q2	3,053	33	6.2	264	1.392
	18,973	Q3	6,031		3.1	134	0.705
Pyrenees	2,809	Q1	985		2.9	121	4.314
	2,809	Q2	3,053		0.9	39	1.392
	2,809	Q3	6,031		0.5	20	0.705
SW France	31,430	Q1	985		31.9	1356	4.314
	31,430	Q2	3,053		10.3	437	1.392
	31,430	Q3	6,031		5.2	221	0.705
Narbonne area	4,600	Q1	985		4.7	198	4.314
	4,600	Q2	3,053		1.5	64	1.392
	4,600	Q3	6,031		0.8	32	0.705
W central France	3,080	Q1	985		3.1	133	4.314
	3,080	Q2	3,053		1.0	43	1.392
	3,080	Q3	6,031		0.5	22	0.705
S-Rhône	5,792	Q1	985		5.9	250	4.314
	5,792	Q2	3,053		1.9	81	1.392
	5,792	Q3	6,031		1.0	41	0.705
Belgium	7,276	Q1	1,006		7.2	308	4.226
	7,276	Q2	1,416	14	5.1	218	3.000
	7,276	Q3	2,025		3.6	153	2.099
Upper Danube	4,654	Q1	1,006		4.6	197	4.226
	4,654	Q2	1,416		3.3	140	3.000
	4,654	Q3	2,025		2.3	98	2.099
NW Czech Rep.	1,216	Q1	3,434		0.4	15	1.238
	1,216	Q2	5,036	19	0.2	10	0.844
	1,216	Q3	7,152		0.2	7	0.594
Danube / Moravia	19,720	Q1	3,434		5.7	244	1.238
	19,720	Q2	5,036		3.9	166	0.844
	19,720	Q3	7,152		2.8	117	0.594
Krakow Area	2,865	Q1	3,434		0.8	35	1.238
	2,865	Q2	5,036		0.6	24	0.844
	2,865	Q3	7,152		0.4	17	0.594
Middle Tisza	2,095	Q1	1,571		1.3	57	2.706
	2,095	Q2	3,468	5	0.6	26	1.225
	2,095	Q3	10,779		0.2	8	0.394
Upper Tisza	2,678	Q1	1,571		1.7	72	2.706
	2,678	Q2	3,468		0.8	33	1.225
	2,678	Q3	10,779		0.2	11	0.394
**Sum Core Area**	***107*,*188***						

Shading indicates CA for which CatA values were combined and/or transferred.

* = summed Core Areas of Asturias, Cantabria / Basque Country.

Turning to the EA supra-regional scale, which combines the spatial data on site density and lithic raw material transport, only a few more sites (88%, 268 out of 304) are covered, lending robustness to the scale of the CAs. Two distinct patterns emerge at the scale of the EAs: the W European EA and the E EA connect several CAs by raw material transport, while all north-central EAs (Belgium, W Czech Republic, upper Danube) only contain one CA each (Fig 2: lower). Density estimates within the north-central EA (S2 Table) are highest in Belgium and the upper Danube with 1.501 and 1.351 P/100 km^2^ respectively. Estimates for the NW Czech Republic lie at 0.208 P/100 km^2^, and for the Eastern EA 0.194 P/100 km^2^. Values for the Western EA are again higher (0.589).

## Discussion

The population estimates presented in this study for the TAC (ranging from 0.059–0.254 P/100 km^2^) are lower than those obtained by other studies; although a methodologically similar approach using site numbers and ethno-historic population densities arrived at a similarly low mean density estimate of 0.168 P/100 km^2^ for entire Europe ([10]:1664). We argue that this is because the TAC focuses on an area of general high population density. If including reported empty areas—such as Southern Iberia and Britain—our estimates would be reduced considerably in comparison. The same argument holds for diachronic estimates presented for several Upper Paleolithic periods using ethno-historic data and a climate envelope modeling approach [12]: much higher densities, 4.4 P/100 km^2^, were predicted in a slightly younger period, around 30 ky BP, with highest densities being observed in Iberia. Even at the scale of CAs, our approach does not arrive at such high densities. Both approaches exclude economically uninhabitable areas from calculations, however, they do not consider scaling effects in ethno-historic density data [46], which were avoided by the present approach using group size data instead. In the same line, we argue that site-distribution patterns, although being subject to frequently discussed biases, must be considered as a source of information on spatial organization too, and settlement intensity should not be averaged across Europe.

The methodological and theoretical differences are important and become even more apparent when we turn to the regional scale, i.e. the estimates for the CA and EA. Of vital interest to the study of human populations are the identification of both the minimum size required for a population to be demographically viable, and the socio-spatial strategies applied to cope with social tensions, low densities or heterogeneously dispersed groups (e.g. [15]). Analyses of ethnographically documented hunter-gatherer population sizes (e.g. [13, 44, 47–49]) show that periodically aggregated groups (Group 3 according to Binford, [44]) range from 100–200 persons. This is corroborated by agent-based modelling [50], where a stable population size of about 150 persons is shown to be demographically viable, i.e. capable of persisting at a specific statistical probability level over several generations. Larger social entities documented in the ethnographic record thus rather allow for buffering stochastic perturbations on larger temporal scales, while groups of 150 persons seem likely to represent viable entities at the scale of several generations. This threshold has been observed in our CA population estimates as well. However, patterns of archaeological relicts do not reflect a moment, a census, in time, and the marked differences of population size and density across Europe and between Core Areas requires further discussion. Insights derived from a large-scale diachronic study of our database and possible effects of distinct mobility and connectedness on the socio-spatial organization are presented in the following sections.

### Diachronic changes during the Aurignacian

To see whether the observed patterns in the population estimates might be related to internal, diachronic changes in human presence on the landscape during the early Upper Paleolithic we generated and compared CAs for Proto / Early Aurignacian assemblages (Phase I) and for assemblages attributed to the later phases of the Aurignacian (Phase II). Since our geostatistical protocol describes density relative to each defined period, a correction of the data for the duration of a phase is not required. We do not, however, present population estimates for each phase, since a temporal subdivision of the raw material data was not consistently possible. This circumstance renders the calculation of population estimates for subphases of the Aurignacian currently impossible. The general tendency, however, shows an increase in CA size and CA number, and a decrease in the maximum distance between sites with a LEC radius dropping from 42 to 35 km (Table 4). These data support an interpretation of the evidence as an increase of the general population size as well as the population density from Phase I to Phase II.

**Table 4:** Main characteristics of the Core Areas calculated for the two phases of the Aurignacian (see text).

	Aurignacian Phase I	Aurignacian Phase II
CA (km^2^)	81,900	128,600
Number of CA	8	10
Kilometre of LEC	42 km	35 km
Number of sites in TAC	87	267
% of sites within CA	74%	79%

**Fig 3:**
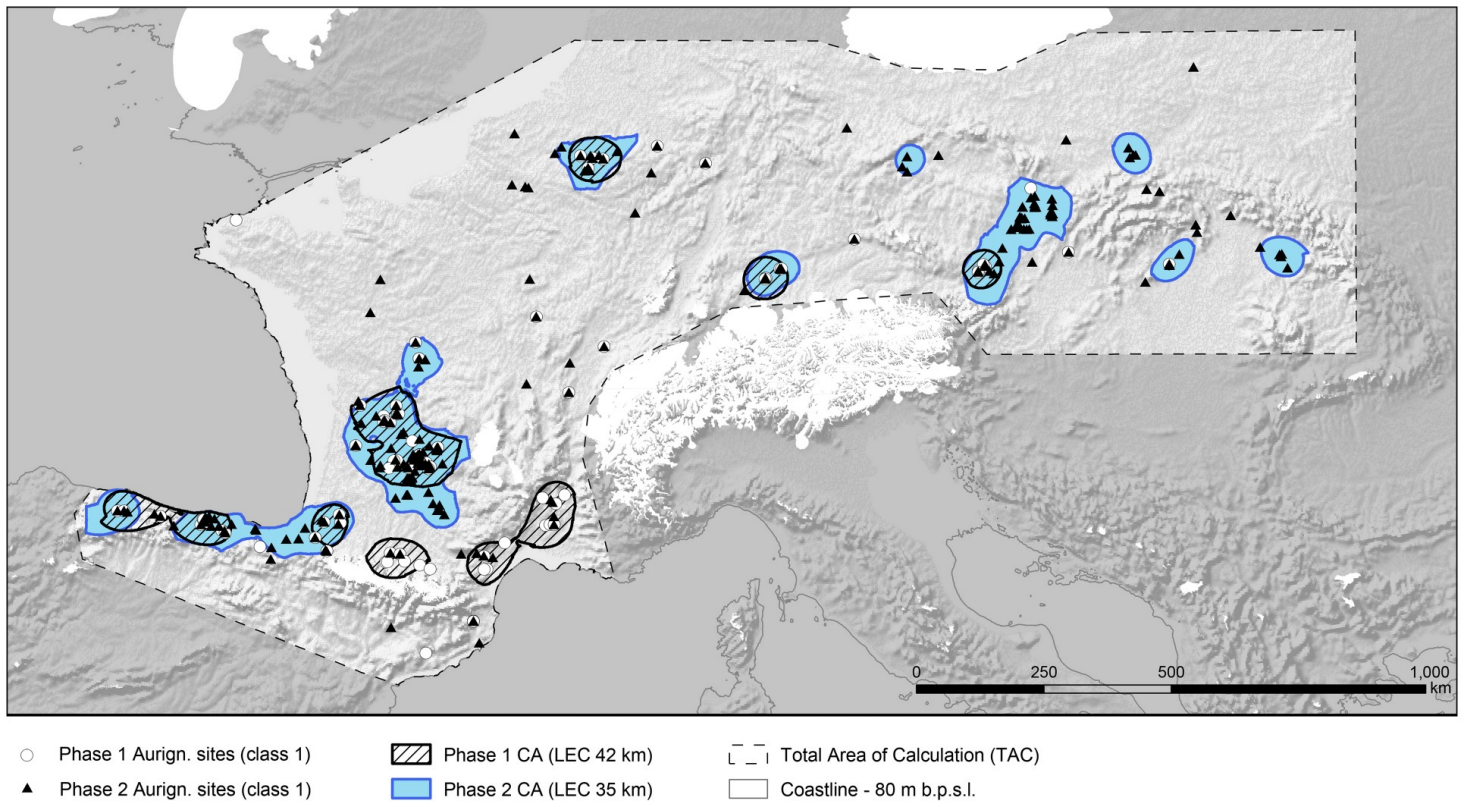
Spatial changes and (dis-)continuity for the occurrence of assemblages attributed to phase I as defined in this study (Proto and Early Aurignacian, white dots) and phase II (assemblages attributed to subsequent phases of the Aurignacian, triangles) within the Total Area of Calculation (TAC). Optimal Isolines calculated separately for each phase reveal distinct spatial patterns of Core Areas during the first (hatched areas) and second phase (blue areas).

For further analysis, and being aware of the uncertainties inherent in the underlying data, we only characterize larger areas as featuring either a) stable / continuous occupation, b) retreat or c) expansion (Fig 3). Important for evaluating the population estimates presented above, the diachronic trends foster four observations. Firstly, CA with large (≥150) population estimates are areas characterized by a clear continuity in occupation during the two phases of the Aurignacian. This supports their interpretation as successfully established and probably viable populations (see also [50]). Most of these CAs also show a marked increase in the extent of the occupied area during the later phase of the Aurignacian, especially in SW France and the Moravian region. Secondly, in contrast, all CAs with estimates <80 persons seem to have experienced either an expansion, i.e. a late occupation during the Aurignacian, or a retreat. In southernmost France, the retreat occurred relative to the overall abundance and distribution of sites, but did not leave the areas empty. Thirdly, the fairly low population estimate of 140 persons for the upper Danube CA increases and clearly expands towards adjacent eastern areas during the early Gravettian [19]. In this instance, the estimate might be explained as evidence of the initial stage of a population that later became well established. Finally, discontinuity in occupation poses a challenge to the method. The study indicates that CA with population estimates <80 must consider internal temporal patterns in occupation history to avoid biases caused by diachronic population fluctuations. At the present state of knowledge, exclusion of these small areas would reduce the overall estimate by about 300 persons. Since all areas contributed people during at least one of the phases with similarly intensive occupations to the overall population habitat, we decided to keep them in our final results (Table 3).

### Spatial patterns related to population movements

The spatial data produced for the diachronic study (Fig 3) also capture information indicating different patterns of human mobility and expansion during the Aurignacian. We do not expect to find patterns produced by individual movements, but patterns resulting from socio-spatial organization. In this sense, we refer to common models on species’ distribution from historical biogeography [51], not disregarding the decisive role of cultural and behavioral conditions shaping the processes (e.g. [52, 53]). We deduce three distinct modes of population movements across landscape: Firstly, large-scale areas—i.e. Great Britain and Iberia south of the Cantabrian Province—with a chronometrically evidenced delayed Aurignacian (or even very early Gravettian) occupation corresponding to Phase II, show an ephemeral but even distribution of sites. This suggests that initial movement of people into these areas was fast and widespread [54], without necessarily establishing successful regional populations “along the way”, as it is often assumed for colonization processes. As noted previously, the perception of unknown territories as well as possible forms of mobility of Pleistocene hunter-gatherers might be without any recent analogues [55–57]. Based on the method at hand, the presence of established populations in southern Iberia is only testified for the early Gravettian in central Portugal [19] and along several coastal regions of southern Iberia for the Solutrean period ([18], cf. also [58]). Thus, the absence of modelled CAs in these regions might actually relate to an initial pioneer “gradient”, which becomes invisible when time-averaged spatial patterns of high densities of human occupation overlay the pattern [59].

Secondly, the diachronic comparison shows that the CAs with large population estimates (≳150) in northern Spain, SW France and in the middle Danube/Moravian region significantly expanded—rather than shifted—the occupied areas into adjacent regions through time. This process of expansive diffusion into formerly unoccupied adjacent areas is most likely expected to have occurred during phases of population increase.

Thirdly, all small and geographically isolated CAs with estimates <80 persons occur in the vicinity of CAs with large population estimates. The distance between the centers of the nearest small and large CA-populations is about 200 km. The distances for the western CAs are slightly lower (mean 180 km) than for the eastern ones (mean 220 km). Based on the low population estimates of the small CAs it can be argued that this pattern results from satellite groups depending on the core population for their viability. Alternative explanations could consider them as seasonally occupied areas. The movement of groups between core- and satellite areas is supported by the repeated transport of raw material (see below). Since our study documents the latter distribution pattern across Western and Eastern European regions, the establishment and maintenance of those provisionally termed “satellite areas” is a widespread and characteristic feature of settlement strategy during the Aurignacian. However, it only becomes widespread during the first phase of the Gravettian, while during the Aurignacian large CA-populations of northern-central Europe, i.e. the Belgium and Upper Danube CAs, are not surrounded by satellite CAs. This allows us to compare—on a broad scale—differences in correlates of mobility and connectedness between the CAs.

### Population connectedness during the Aurignacian

#### Raw material transport

Evidence of lithic raw material transport is of vital interest in the understanding of territorial organization, mobility and the interconnectedness of prehistoric people [60–63]. For the early Upper Paleolithic, three areas provided a reasonable amount of data: N Spain/SW France, Belgium, and the middle Danube/Moravian region. Although each region suffers from specific problems related to the methodological robustness of data (attribution of archaeological finds to raw material sources, identification of source locations, e.g. [60, 64]), they offer initial broad-scale insights into aspects such as frequencies, distances and directions of transported lithic raw material.

**Fig 4:**
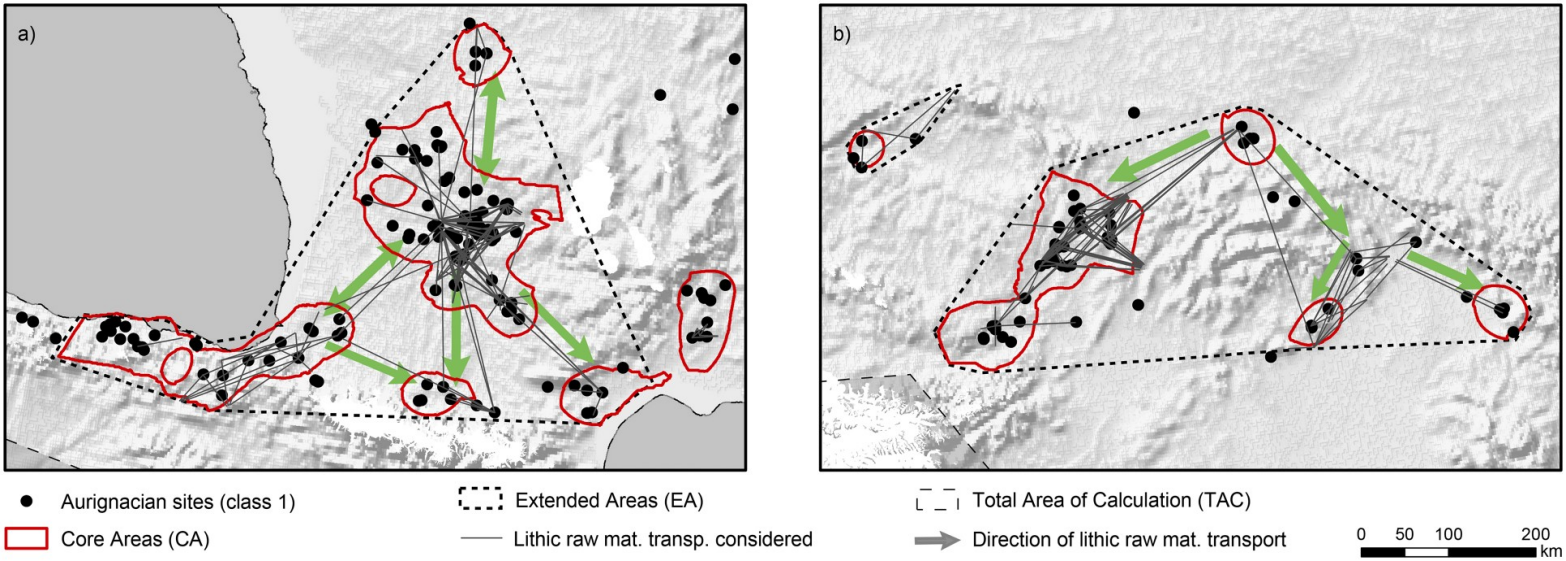
Direction of repeated lithic raw material transport between Core Areas for the western (left) and eastern (right) Extended Area (for an overview on map-sections see Fig 2). Evidence of uni- or bidirectional transport is indicated by arrows.

Two observed patterns are highlighted: Firstly, sources of exploited raw materials are overwhelmingly often located within CAs in the western and eastern EAs (Fig 4). Only few instances document the use of sources from outside a CA: this is the case for CAs of central northern Europe as well as the Upper and Lower Tisza region. Secondly, although most of the used raw material was sourced and discarded within the same CA, transport of raw materials into other CAs is also frequently observed. Again, this is restricted to the western and the eastern EAs (Fig 4). Transport of raw material in the north-central EAs does not connect different CAs.

These observations have implications for our approach: The first observation adds enormous significance and robustness to the concept and spatial scale of the CA as a valuable analytical tool. It implies that the CA, defined solely by the density of archaeological sites (maximum distance of 58 km between sites), has significance for the behavior of raw material sourcing, too. In several instances, procurement occurred at sources which are located just at the border of the CA, i.e. exploited sources are right at the “maximum distance”—between and from sites—and not within dense clusters of sites. This holds true for example in N Spain for Trevino and Urbasa raw material; in SW France for jasper Hettangien, Grain de Mil and Grand-Pressigny flint; in Belgium for the silex of Haunaut and phtanite of Mousty; and in the Moravian area for the radiolarite of Váh and the commonly referenced region for erratic Baltic flint [60].

However, this correlation of two–*a priori* expectedly related—behavioral patterns is only confirmed for the Aurignacian period, while for all later Upper Paleolithic periods site-density based CAs and the location of exploited raw material sources are spatially less related [16, 18, 19]. The patterns result from multi-causal processes governed by cultural, technological and economic needs, which are impossible to disentangle at a large scale. It does, however, indicates that hunter-gatherers of the Aurignacian—more than during later phases of the Upper Paleolithic—arranged raw-material procurement in accordance with their commonly inhabited areas, and vice versa. Observations on site organization and inferred mobility patterns repeatedly suggest residential mobility for the central regions, with a lack of long-distance logistical camp sites (see [65] and references therein). The above mentioned outliers concern the Tisza region—where obsidian and radiolarite sources are located at the geographical midpoint between two CAs—and the north-central CAs (Figs 2 and 4).

The second observation relates to the repeated connection between CAs by raw material transport. North-central CAs remain unconnected, while both the W and E EAs experience high connectedness of CAs by raw material transport. In the E EA we only see unidirectional flint transport from the Kraków area into south-western and south-eastern direction, a separation already recognized by Kozlowski [66]. Evidence of transport further south into the Danube region is scarce and difficult to determine [67]. Flint imports to the Slovakian settlements provide evidence for the importance of the Carpathian passes in social networks. A similar role for the Moravian Gate was suggested [68], but so far we do not detect evidence for an established population north of the Moravian Gate before the late and final Magdalenian period [16, 40].

Evidence of repeated bidirectional raw material transport is so far only available within the W EA (Fig 4; see also [62]:Fig 3.6) and connects CAs characterized by continuous occupation (W central France) as well as large population sizes (N Spain and SW France). The distinct bidirectional connections documented between central/south-western France and northern Spain hint at a different role of Cantabria and the Basque country within the overall population dynamics of Western Europe, likely oscillating between a viable core- and dependent satellite population or seasonal habitation, respectively. CAs with small population estimates (<80 persons) see unidirectional raw material import. The CA of the lower Rhône valley remains isolated, although evidence for economically relevant connections to the Narbonne area are now emerging [65]. On a longer perspective, the developments during the Gravettian period indicate a close relationship between this region and northern Italy, a social bond that might have already become established during the Aurignacian.

#### Personal ornaments

**Fig 5:**
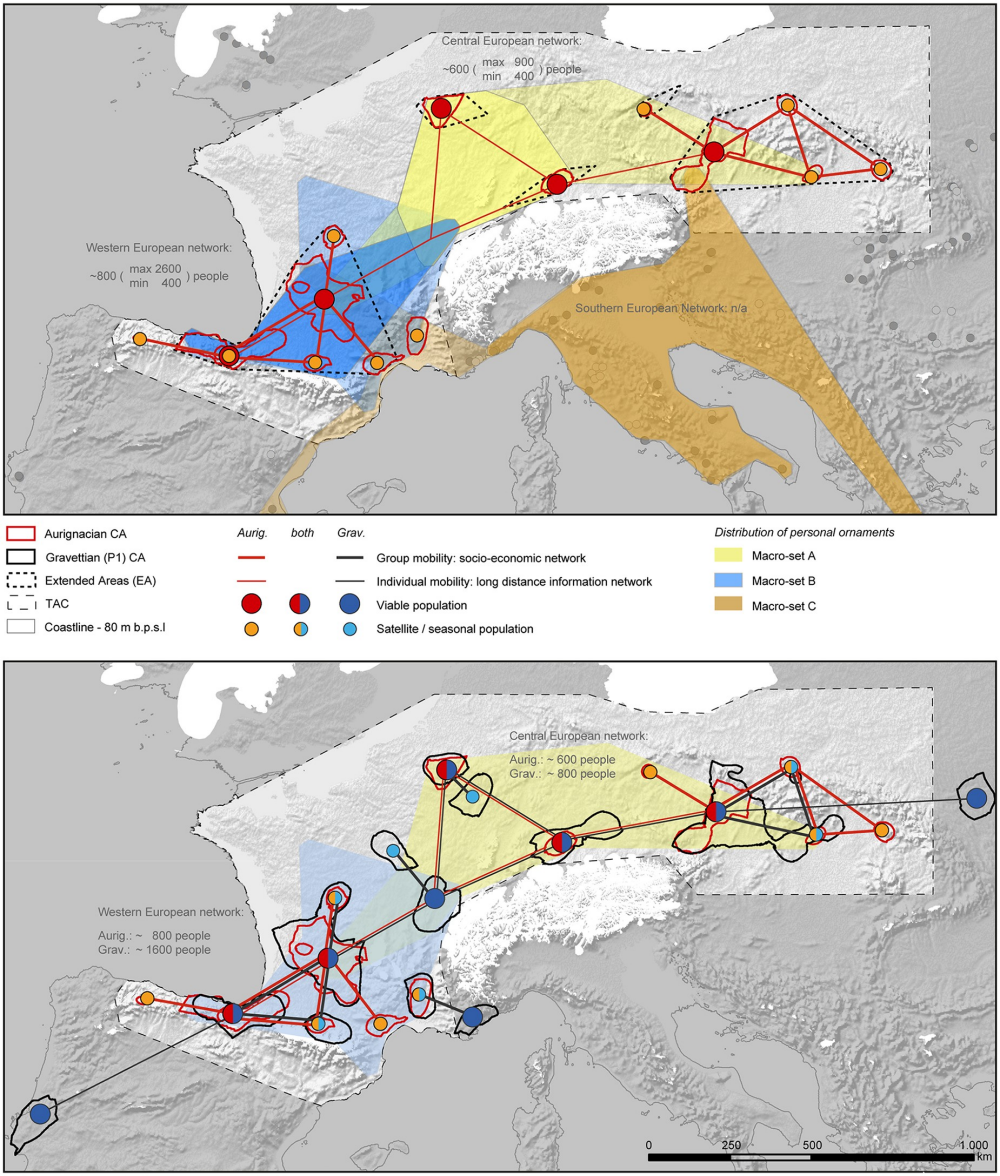
Synchronic and diachronic representation of the model on socio-spatial organization. Upper image: Manifestation during the Aurignacian. Location of Core Areas (CA) and demographic estimates from this study are indicated by circles. Mapping of “macro-sets” identified by seriation (after: [69], colored areas = macro-set-specific ornament types, light shaded areas = ornament types shared by two macro-sets). Lower image: Diachronic comparison of the socio-economic and long-distance information network of the Aurignacian (red lines, this study) and the Gravettian (black, after: [19]), based on CAs and population estimates. The pattern of viable and seasonal/satellite populations becomes adopted throughout the Total Area of Calculation (TAC); new evidence emerges in central Europe and beyond the TAC.

Additional information is obtained from the spatial patterning of beads and other types of personal ornament items across Europe during the Aurignacian [69]. Already introduced as reflecting the ethnolinguistic diversity of the earliest Upper Paleolithic populations of Europe ([69]:1105), such items of personal adornment provide important insights into social interaction. We adopted the classificatory categories and the macro-sets identified by seriation from the original publication on the topic ([69]:Fig 5). Three macro-sets are distinguished. Their spatial extents cover Northern Europe (macro-set A), Spain and south-western France (macro-set B), and Greece, Austria, Italy and the Rhône valley (macro-set C). We separately mapped the distribution of ornament types specific to each macro-set (Fig 5: upper, dark shaded areas), and of types which are shared between macro-sets (Fig 5: upper, light shaded areas). As already noted by the authors, the Spanish-French macro-set B shares several ornament types with both A and C, while macro-set A and C share none.

Only two marginal regions of the TAC defined in this study overlap with macro-set C: south-eastern France and north-central Austria. Vanhaeren and d’Errico’s seriation results indicate relationships between these regions and the Mediterranean/Balkan region. This observation correlates extremely well with raw material studies indicating a close connection of the French Rhône- to the Italian Po-valley [70], and between the north-central Austrian sites and the Moravian region [60, 64, 67]. Both areas play decisive roles within models on the expansion of the earliest Aurignacian (e.g. [2]:Fig 3), irrespective of the temporal and cultural relationships of Proto- and Early Aurignacian Phases.

Occurrences of ornaments specific of macro-set B follow the outline of the Extended Area of N Spain and SW France, with extensions to the North, i.e. into the lower Loire and the Bourgogne area. Importantly, with regards to socio-spatial organization, a clear overlap of characteristic ornaments from macro-set A and B is evidenced in the Bourgogne area, connecting the remaining CAs of central and Eastern Europe. The overlap describes the geographical midpoint between three major CAs defined by our demographic analysis. Evidence of Macroset A extending towards the eastern Extended Area is scarce and little understood in terms of temporal resolution. At this scale, the spatial outlines of the Macrosets A and B of personal ornaments allows the delineation of two distinct global networks (*sensu* Gamble, [1]:51) for which the demographic estimates predict fairly similar numbers of people—800 and 600—although very different ranges and densities (Fig 5: upper).

As an aside, we observed a strong correlation between the recorded presence of ornaments in Vanhaeren and d’Errico’s database and the attribution of sites to the Early Aurignacian in ours. The use of the split-based points as a *fossil-directeur* for Early Aurignacian assemblages [6, 21, 71] might have actively generated some of the chronological as well as spatial patterns discussed here.

## Socio-spatial organization and population dynamics of the early Upper Paleolithic

The new results and data presented and discussed within the previous sections allow for multiple and regionally varying explanatory scenarios, although it is not the scope of this paper to test these hypotheses on regional grounds. As for the large-scale approach of this study, which encompasses the above-defined TAC, we propose a general socio-spatial organization for the Aurignacian that rests upon our finding of successfully established, continuous and viable populations (≥150 persons) being established across Europe and separated by a distance of around 400 km—as the crow flies—from each other (Fig 5: upper). This observation leads us to argue for a “social carrying capacity” of human groups inhabiting a landscape. This social carrying capacity is shaped by cognition and sociality of humans and expected to be expressed in the socio-spatial patterns. This concept clearly differs from its economic namesake, although equifinality or adaptive processes of both social [1, 72] and economic realms could lead to the observable patterns [73]. Exploration of the conceptual and practical distinction between social and economic carrying capacities in the interpretation of large scale archaeological patterns is a worthwhile avenue of further research. Clarification of the repeatedly observed “mismatches” between predicted (based on paleo-environmental and/or ethno-historic data) and observed human presence [18, 12, 14] or of changes in the “attractiveness” of landscapes [57] could be one of its promising outputs.

From the perspective of the socio-spatial organization, our site-density analysis and demographic estimates also repeatedly indicate the presence of non-viable populations in areas at about 200 km distance from the centers of viable populations. Based on the very low population estimates and the repeated and economically significant long-distance raw-material transports between the core and “satellite” areas, we favor an interpretation of the latter as the extent of the annual range of group-mobility from the core areas (see also [62]). Such group fission-and-fusion processes over long distances have already been suggested by Bordes and colleagues for southern France with regard to the seasonality of resources [74]. Our scaled demographic estimates additionally indicate that this spatial pattern does not occur throughout Europe, but rather in contexts of combined viable populations *and* spatially extensive Core Areas. This would not only be beneficial to cope with distinct (and changing) environments, as Bordes et al. [74] pointed out for SW France, but would also enable people to maintain the frequently mentioned long-distance contacts during the Aurignacian across Europe. From a broader behavioral perspective, such large scale group mobility might also reflect a generalized and transferable system of landscape learning [53] inherent to the first anatomically modern human societies in Europe.

As such, seasonal fission-fusion behavior over long distances could relate to a specific socio-spatial organization during the Aurignacian, while at the same time the reasons for this pattern could relate to the proposed population increases which are compensated for by adjusting mobility at regional grounds [15, 48, 75, 76]: to maintain group size and to avoid social tension, new lower-level groups have to be established [48]. The particular pattern of connectivity apparent for the north-central CAs, which share similar unique personal ornaments but no connecting raw-material transports between CAs, could relate to a distinct organization of mobility, with less long-distance residential mobility of groups and rather more long-distance mobility of individuals across the region.

From a broader diachronic perspective, during the early phase of the Gravettian, a sort of multifactorial maximum is reached—expressed by demographic, cultural and social features (e.g. [1, 77]). The socio-spatial organization of the Aurignacian becomes consolidated across Europe (Fig 5: lower). Populations during the early Gravettian [19] show a clear increase in size (from 1,500 to 2,800 people) and density (from 0.103, TAC = 1,5 million km^2^, to 0.139, TAC = 2 million km^2^) compared to the Aurignacian. This consolidation of the socio-spatial network during the early Gravettian is best exemplified by a new viable population in the Bourgogne area where Aurignacian ornament distributions already indicated a contact zone between the SW and North-central European populations. New satellite/seasonal areas here and around Belgium emerge. The separation between SW- and SE-France becomes more pronounced by the disappearance of the Narbonne CA. A clear spatial expansion of a CA into adjacent regions is observed for the Upper Danube valley, supporting its function for a fully established, viable population. No clear break between the two cultures, i.e. a regional development of the Gravettian, has been proposed here based on lithic studies [77–79], although organic artefacts indicate a different pattern [80]. In the Eastern EA, where contacts seem to become intensified between middle Danube and upper Tisza regions, patterns indicate extensive population diffusion. This observation supports the notion that the clustered evidence of Aurignacian sites reflects historic reality and not preservation biases [81]. A glance beyond the borders of the TAC towards Northern Italy and the Balkan region also supports a spatial continuation of the observed socio-spatial pattern, although currently the density of securely dated or attributable sites is very low and biased, and therefore would only allow our methodological protocol to detect CAs with higher LEC-distances than within the current TAC.

The proposed scenario of socio-spatial organization across centers and satellite/seasonal areas of the human population predicts high mobility with long distant contacts established between populations. It also assumes adaptation at the level of groups to different ecological habitats and even biomes during seasonal rounds. Whether this would already reflect an adaptation to “megapatches” [82] depends on the degree of environmental diversity and the spatial scale under consideration. The knowledge of several gross environmental categories would provide fundamental advantages to cope with environmental changes and allow for easy diffusion of people and ideas across the subcontinent [53]. In this regard, our regional demographic data do also provide a detailed framework for contextualizing genetic evidence. New data and scenarios were recently proposed for the Upper Paleolithic [83, 84] which not yet consider such detailed data on population density, regional varying population increase, patterns of interconnectedness, abandonment of regions, or extinctions. The fixation of markers within small and low density populations occurs faster and with very high amplitude of success and failure. While beyond the scope of this paper, it will be worthwhile researching the effects of drift in conjunction with our proposed regional population dynamics [85].

The large-scale synchronic and diachronic observations presented allow for the development of hypotheses of culturally inherited principles of socio-spatial organization which seem to persist over long time spans and through distinct climatic and environmental contexts. Our intention was not to provide detailed local or regional occupation histories. Further analysis and testing of the proposed regional scenarios, at the scales of CAs and EAs as informed starting points, will improve our understanding of the socio-spatial organization of hunter-gatherers in western and central Europe during the Upper Paleolithic.

## Supporting information

S1 Fig Schematic illustration of the spatial data and geostatistical procedures applied in the present study. Supplementary to Table 1.
(TIF)

S2 Fig Area increase (km^2^) within the Isolines set in relation to the size (km) of the Largest Empty Circle (LEC) radius. A first maximum followed by a pronounced decrease can be identified at 29 km.
(TIF)

S1 Table Assemblages with calculated raw material Catchment Areas. The assemblages with calculated raw material Catchment Areas (CatA), and CatA circumference (Circ), are sorted based on assigned Region (cf. Table 2) and CatA size. CatA <500 km^2^ were excluded, as well as assemblages located outside the Total Area of Calculation (TAC).
(DOCX)

S2 Table Population numbers and densities calculated for the Extended Areas (cf. Fig 2). The numbers of sites for each EA are given in brackets. For abbreviations see Table 1.
(DOCX)

S1 File Defining the Total Area of Calculation (TAC). Supporting information on the criteria used to define the extent of the TAC. References of the Supporting Information.
(DOCX)
