# Supplementary figures and images for: Population dynamics and socio-spatial organization of the Aurignacian: Scalable quantitative demographic data for western and central Europe
Source: PLoS One. 2019 Feb 13;14(2):e0211562. doi: 10.1371/journal.pone.0211562 (PMC6373918; doi:10.1371/journal.pone.0211562)

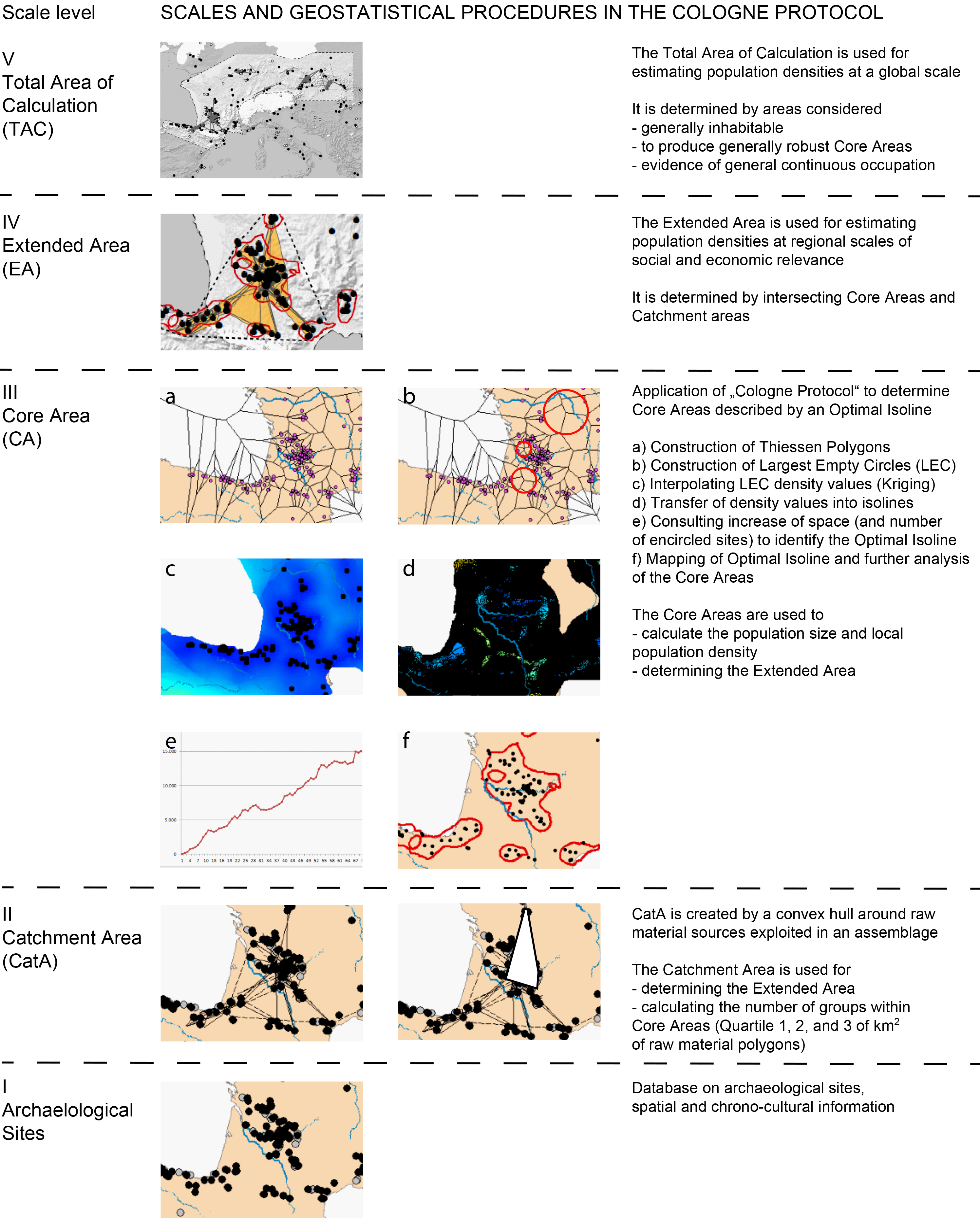

Supplement: S1 Fig — Supplementary to Table 1. (TIF) [file pone.0211562.s001.tif]

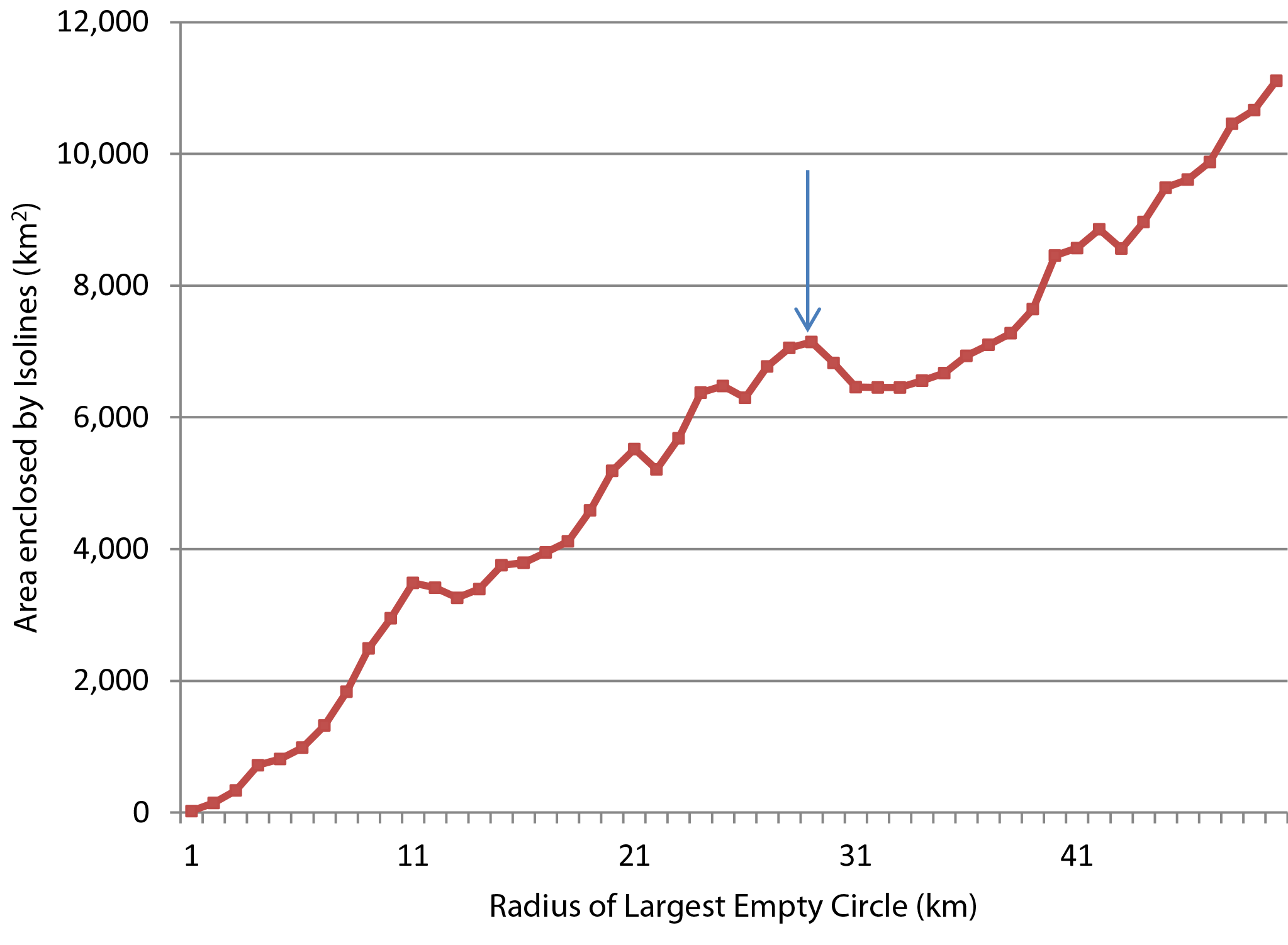

Supplement: S2 Fig — A first maximum followed by a pronounced decrease can be identified at 29 km. (TIF) [file pone.0211562.s002.tif]
