# Supplementary material for: Population dynamics and socio-spatial organization of the Aurignacian: Scalable quantitative demographic data for western and central Europe
Source: PLoS One. 2019 Feb 13;14(2):e0211562. doi: 10.1371/journal.pone.0211562 (PMC6373918; doi:10.1371/journal.pone.0211562)
Supplement: S1 Table — The assemblages with calculated raw material Catchment Areas (CatA), and CatA circumference (Circ), are sorted based on assigned Region (cf. Table 2) and CatA size. CatA <500 km2 were excluded, as well as assemblages located outside the Total Area of Calculation (TAC). (DOCX) [file pone.0211562.s003.docx]

| **Area**  Name of Site | Country | CatA (km^2^) | Circ (km) | **Region** | **Reference** | |
| --- | --- | --- | --- | --- | --- | --- |
| **N Spain** |  |  |  |  |  | |
| Cova Gran | Spain | 111 | 47 | **excl** | [10] | |
| Viña, La | Spain | 257 | 105 | **excl** | [11] | |
| Otero, Cueva del | Spain | 284 | 118 | **excl** | [12] | |
| Labeko Koba | Spain | 2843 | 254 | **NSpain/Pyrenees** | [13] | |
| Ekaïn | Spain | 3282 | 280 | **NSpain/Pyrenees** | [14] | |
| Aitzbitarte III | Spain | 3914 | 343 | **NSpain/Pyrenees** | [12] | |
| Hyènes, Grotte des | France | 7018 | 708 | **NSpain/Pyrenees** | [15] | |
| Tutto de Camayot | France | 20344 | 616 | **NSpain/Pyrenees** | [15] | |
| **SW France** |  |  |  |  |  | |
| Faurélie, La; ML-Aur | France | 166 | 70 | **excl** | [16] | |
| Poisson | France | 185 | 77 | **excl** | [16] | |
| Cellier | France | 198 | 83 | **excl** | [16] | |
| Cellier c. sup; ML-Aur | France | 201 | 81 | **excl** | [16] | |
| Le Facteur niv. 21 | France | 202 | 84 | **excl** | [17] | |
| Facteur, Le; ML-Aur | France | 204 | 82 | **excl** | [16] | |
| Laouza, La | France | 278 | 68 | **excl** | [18] | |
| Barbas III | France | 312 | 120 | **excl** | [19] | |
| Esquicho Grapaou | France | 376 | 83 | **excl** | [18] | |
| Laburlade; ML-Aur | France | 534 | 104 | **SW France** | [16] | |
| Dau, La | France | 595 | 190 | **SW France** | [16] | |
| Coumba-del-Boui¨tou; ML-Aur | France | 647 | 172 | **SW France** | [16] | |
| Pepeyrou | France | 656 | 108 | **SW France** | [16] | |
| Lasgardes-Haut | France | 671 | 176 | **SW France** | [16] | |
| Peyrony; ML-Aur | France | 805 | 117 | **SW France** | [16] | |
| Lartet | France | 813 | 191 | **SW France** | [16] | |
| Mandrin, Grotte | France | 885 | 169 | **SW France** | [20] | |
| Las Pelenos; ML-Aur | France | 985 | 132 | **SW France** | [16] | |
| Toulousette | France | 1033 | 178 | **SW France** | [16] | |
| Estelès- Bulit | France | 1381 | 168 | **SW France** | [16] | |
| Chanlat c. sup; ML-Aur | France | 1798 | 203 | **SW France** | [16] | |
| Roches, Les | France | 2369 | 455 | **SW France** | [21] | |
| Rois, Les | France | 2555 | 255 | **SW France** | [22] | |
| Chanlat c. inf; ML-Aur | France | 3025 | 237 | **SW France** | [16] | |
| Coumba-del-Boui¨tou | France | 3053 | 239 | **SW France** | [16] | |
| Bassaler (Nord?) | France | 3056 | 236 | **SW France** | [16] | |
| Font-Yves | France | 3061 | 237 | **SW France** | [16] | |
| Bos del Ser; ML-Aur | France | 3063 | 238 | **SW France** | [16] | |
| Pataud, Abri | France | 4606 | 384 | **SW France** | [23] | |
| Roc de Combe c. 9,7c,7b,7a | France | 5942 | 420 | **SW France** | [16, 24] | |
| Isturitz | France | 6031 | 407 | **SW France** | [25] | |
| Régismont le Haut | France | 6096 | 568 | **SW France** | [15, 24, 26] | |
| Caminade | France | 6794 | 508 | **SW France** | [24] | |
| Moulinière, La | France | 8572 | 364 | **SW France** | [16] | |
| Hui | France | 9007 | 509 | **SW France** | [16] | |
| Le Piage c. K,J,GI | France | 10279 | 543 | **SW France** | [16, 24] | |
| Dufour; ML-Aur | France | 12063 | 577 | **SW France** | [16, 24, 26] | |
|  |  |  |  |  |  | |
| **N-Central Europe** |  |  |  |  |  | |
| Fonds de Forêt, Grotte de | Belgium | 89 | 39 | **excl** | [16] | |
| Altwies-Laangen Aker | Luxemburg | 262 | 75 | **excl** | [27] | |
| Sirgenstein V-VI | Germany | 425 | 87 | **excl** | [16] | |
| Betche aux Rotches | Belgium | 1006 | 163 | **Belgium/Ger** | [16] | |
| Diable, Trou du | Belgium | 1479 | 169 | **Belgium/Ger** | [16] | |
| Trou Magrite | Belgium | 1979 | 182 | **Belgium/Ger** | [16] | |
| Renard, Trou du | Belgium | 1625 | 180 | **Belgium/Ger** | [16] | |
| Princesse Pauline, Grotte de la | Belgium | 616 | 146 | **Belgium/Ger** | [16] | |
| Prince, Grotte du | Belgium | 1205 | 157 | **Belgium/Ger** | [16] | |
| Goyet | Belgium | 2040 | 210 | **Belgium/Ger** | [16, 28] | |
| Cave, Grotte de la | Belgium | 1353 | 179 | **Belgium/Ger** | [16] | |
| Lommersum | Germany | 577 | 179 | **Belgium/Ger** | [16] | |
| Wildscheuer c. III | Germany | 2577 | 305 | **Belgium/Ger** | [16] | |
| Nesuchyne | Czech Rep. | 2280 | 294 | **NW-Czech Rep.** | [29] |  |
| Hradsko | Czech Rep. | 2283 | 254 | **NW-Czech Rep.** | [29] | |
| Vogelherd V-VI | Germany | 1006 | 209 | **Upper Danube** | [16, 30] | |
| Hohlenstein-Stadel V | Germany | 972 | 211 | **Upper Danube** | [16, 30] | |
| Geissenklösterle - Level IIIb-IId | Germany | 5216 | 617 | **Upper Danube** | [16, 30] | |
| Geissenklösterle - Level IIb-IIn | Germany | 5257 | 617 | **Upper Danube** | [16, 30] | |
|  |  |  |  |  |  | |
| **Middle Danube** |  |  |  |  |  | |
| Krepice | Czech Rep. | 112 | 50 | **excl** | [16] | |
| Alberndorf | Austria | 1011 | 156 | **Middle Danube** | [31] | |
| Stránská Skála IIIa | Czech Rep. | 1046 | 232 | **Middle Danube** | [16] | |
| Vedrovice II | Czech Rep. | 1050 | 271 | **Middle Danube** | [16] | |
| Tvarozná | Czech Rep. | 4584 | 312 | **Middle Danube** | [16] | |
| Diváky | Czech Rep. | 4893 | 314 | **Middle Danube** | [16] | |
| Brodek I | Czech Rep. | 4953 | 317 | **Middle Danube** | [16] | |
| Podstránská | Czech Rep. | 4958 | 319 | **Middle Danube** | [16] | |
| Malomerice-Borky II | Czech Rep. | 5036 | 318 | **Middle Danube** | [16] | |
| Malomerice-Obciny | Czech Rep. | 5068 | 316 | **Middle Danube** | [16] | |
| Ondratice II | Czech Rep. | 5559 | 372 | **Middle Danube** | [16] | |
| Galgenberg, Stratz. | Austria | 6057 | 450 | **Middle Danube** | [32] | |
| Lhotka | Czech Rep. | 7037 | 442 | **Middle Danube** | [16] | |
| Zlutava I | Czech Rep. | 7111 | 446 | **Middle Danube** | [16] | |
| Kvasice I | Czech Rep. | 7194 | 445 | **Middle Danube** | [16] | |
| Klobouky | Czech Rep. | 10151 | 567 | **Middle Danube** | [16] | |
| Urcice Golst | Czech Rep. | 11176 | 567 | **Middle Danube** | [16] | |
| Slatinky | Czech Rep. | 12580 | 569 | **Middle Danube** | [16] | |
|  |  |  |  |  |  | |
| **Tisza Area** |  |  |  |  |  | |
| Kechnec | Slovakia | 1195 | 269 | **Theiß Area** | [16] | |
| Barca II | Slovakia | 1571 | 222 | **Theiß Area** | [16] | |
| Istállóskö | Hungary | 3468 | 271 | **Theiß Area** | [16] | |
| Tibava | Slovakia | 10779 | 607 | **Theiß Area** | [16] | |
| Barca I | Slovakia | 11220 | 562 | **Theiß Area** | [16] | |
|  |  |  |  |  |  | |
| **Italy (outside TAC)** |  |  |  |  |  | |
| Riparo Mochi | Italy | 21930 | 749 | **excl** | [33] | |
| Lemignano | Italy | 921 | 415 | **excl** | [34] | |
| Fumane, Grotta di | Italy | 718 | 120 | **excl** | [35] | |
| La Fabbrica | Italy | 1217 | 368 | **excl** | [17] | |
| Riparo di Fontana Nuova | Italy | 591 | 224 | **excl** | [34] | |
