# Supplementary material for: Population dynamics and socio-spatial organization of the Aurignacian: Scalable quantitative demographic data for western and central Europe
Source: PLoS One. 2019 Feb 13;14(2):e0211562. doi: 10.1371/journal.pone.0211562 (PMC6373918; doi:10.1371/journal.pone.0211562)
Supplement: S2 Table — The numbers of sites for each EA are given in brackets. For abbreviations see Table 1. (DOCX) [file pone.0211562.s004.docx]

| **Extended Area** | |  | **Extended Area size (km^2^)** | **Persons (n)** | **Pop Density p/100 km^2^** |
| --- | --- | --- | --- | --- | --- |
|  |  |  |  | 2627 | 1.825 |
| Western EA | N-Spain / SW-France | (166) | 143946 | 848 | 0.589 |
|  |  |  |  | 429 | 0.298 |
|  |  |  |  | 308 | 2.115 |
|  | Belgium | (14) | 14542 | 218 | 1.501 |
|  |  |  |  | 153 | 1.050 |
|  |  |  |  | 15 | 0.305 |
| N-Central EAs | NW-Czech Rep. | (4) | 4942 | 10 | 0.208 |
|  |  |  |  | 7 | 0.146 |
|  |  |  |  | 197 | 1.903 |
|  | Upper Danube | (9) | 10336 | 140 | 1.351 |
|  |  |  |  | 98 | 0.945 |
|  |  |  |  | 409 | 0.318 |
| Eastern EA | Eastern EA | (63) | 128361 | 249 | 0.194 |
|  |  |  |  | 153 | 0.119 |
|  |  |  |  |  |  |
|  |  |  |  | **3555** | 1.177 |
|  | **Sum Extended Areas** | (256) | **302126** | **1465** | 0.485 |
|  |  |  |  | **840** | 0.278 |
