# Supplementary material for: Population dynamics and socio-spatial organization of the Aurignacian: Scalable quantitative demographic data for western and central Europe
Source: PLoS One. 2019 Feb 13;14(2):e0211562. doi: 10.1371/journal.pone.0211562 (PMC6373918; doi:10.1371/journal.pone.0211562)
Supplement: S1 File — Supporting information on the criteria used to define the extent of the TAC. References of the Supporting Information. (DOCX) [file pone.0211562.s005.docx]

### Supporting Information File

**Reviewing and Testing the dataset and defining the Total Area of Calculation**

To identify a suitable Total Area of Calculation (TAC) for the present context, biases of the database and distribution patterns must be discussed. Our aim is to identify a TAC in which currently available data allow robust population estimates for the Aurignacian.

Firstly, we calculated and compared the OI-output for sites considered ‘securely’ (class 1) as well as for a dataset including assemblages whose attribution has been contested or still being open to discussion (class 2). This is to test the robustness of the OI: areas with highly variable outputs are most likely prone to future changes in site distribution patterns, e.g. by re-attributions of assemblages and new chronometric dating. Since it is impossible at this stage to eliminate factors leading to discrepancies between the two datasets, the only solution is to exclude such areas from the present calculation.

Secondly, we checked for information and current models on the settlement history of each region within the TAC from the literature. On a continental scale, the radiocarbon dates suggest a continuous occupation of central Europe during the period defined for this study. This picture is contrasted by evidence from neighbouring regions: For Great Britain, the current model proposes a resettlement of the southern part starting around 37 ky cal BP, coinciding with the maximum of Greenland Interstadial 8 [**1**]. Similarly, the absence of Proto and Early Aurignacian sites in southern Iberia [**2**] as well as contested attributions of Iberian assemblages along the Mediterranean coast to later phases of the Aurignacian [**3**] – suggests only restricted temporal occupation of these regions, too. The initial resettlement of the latter probably took place around 35 ky cal BP [**2**].

For Italy and the Balkan region chronometric data suggest a continuous occupation, which is supported by – although very few – evenly distributed sites across the regions (**Fig. 1**). However, the abundance of sites with contested attributions is high. This observation relates on the one hand to a scarcity of chronometric dates for frequently occurring open-air sites, and on the other hand to research traditions of typological classifications, which created several facies, sub-facies, transitional industries and – as a consequence – an abundance of assemblages classified as ‘auregnacoid’ in both regions [**4, 5**]. Indistinct ‘aurignacoid’ assemblages and collections from the Balkan and adjacent regions have been variably attributed to the Aurignacian or Aurignacian V (‘Aurignacien tardif’, cf. [**6]**; ‘Late Aurignacian’ [**7**], ‘Epiaurignacian’ [**8**]) – a terminological pitfall that has already troubled several large scale projects and limited the value of their datasets. Recently, several of these assemblages have been chronometrically dated to the Last Glacial Maximum (see [**7]**:482; [**8]**). Geochronological estimations in Romania even suggest Tardiglacial ages (see references in [**9]**:176). Thus, research history of these regions as well as the scarcity of well dated assemblages most likely created a biased distribution of sites and were therefore excluded from the TAC.

**S1 Fig.** Schematic illustration of the spatial data and geostatistical procedures applied in the present study. Supplementary to Table 1.

**S2 Fig.** Area increase (km^2^) within the Isolines set in relation to the size (km) of the Largest Empty Circle (LEC) radius. A first maximum followed by a pronounced decrease can be identified at 29 km.

S1 Table. Assemblages with calculated raw material Catchment Areas. The assemblages with calculated raw material Catchment Areas (CatA), and CatA circumference (Circ), are sorted based on assigned Region (cf. Table 2) and CatA size. CatA <500 km^2^ were excluded, as well as assemblages located outside the Total Area of Calculation (TAC).

S2 Table. Population numbers and densities calculated for the Extended Areas (cf. Fig. 2). The numbers of sites for each EA are given in brackets. For abbreviations see table 1.

**References used in the Supplementary Information (Text and Tables)**

1. Dinnis R. The timing of Aurignacian occupation of the British Peninsula. Quartär 2012;59: 67–83.
2. Zilhão J. Chronostratigraphy of the Middle-to-Upper Paleolithic Transition in the Iberian Peninsula. Pyrenae 2006;37: 7–84.
3. De la Peña P, Vega Toscano G. The Early Upper Palaeolithic puzzle in Mediterranean Iberia. Quartär 2013;60: 85–106.
4. Mussi M. Earliest Italy. An overview of the Italian Paleolithic and Mesolithic. New York: Springer; 2001.
5. Palma di Cesnola A. Il paleolitico inferiore e medio in Italia. Firenze, Museo Fiorentino di Preistoria; 2001.
6. Bosselin B,Djinjian F. Une révision du solutréen de Laugerie-Haute et le problème des transitions Gravettien-Solutréen-Badegoulien en Aquitaine, Bulletin de la Société préhistorique Française 1997;94(4): 443-454.
7. Zwyns N. La problématique de l’Aurignacien tardif dans la zone des steppes nord pontiques. Anthropologie 2004;108: 471–493.
8. Mlejnek O. Interdisciplinary Research on the Moravian Upper Palaeolithic in the New Millennium (2001- 2013). Interdisciplinaria Archeologica 2013;4(2): 193–204.
9. Anghelinu M, Niţă L. What’s in a name: The Aurignacian in Romania. Quat Int 2014;351: 172–192.
10. Roy-Dunyer M, Tarriño A, Benito-Calvo A, Mora R, Martínez-Moreno J. Aprovisionamiento de sílex en el Prepirineo oriental durante el Paleolítico superior antiguo: el nivel arqueológico 497C Cova Gran (Santa Linya, Lleida). Trabajos de Prehistoria 2013;70: 7–27.
11. Santamaría Álvarez D. La transición del paleolíthico medio al superior en Asturias. El Abrigo de la viña (la Manzaneda, Oviedo) y la cueva de el Sidrón (Borines, Piloña). PhD Thesis, Universidad de Oviedo; 2012.
12. Rios J, Maillo-Fernandez J, de la Peña P. El final del Auriñaciense y el comienzo del Gravetiense en la región cantábrica: una visión tecno-tipológica. In : Heras Martín C, Lasheras JA, Arrizabalaga A, Rasilla M, editors. Pensando el Gravetiense. Madrid: Museo Nacional y Centro de Investigación de Altamira Monografías 23; 2013. pp 369–382.
13. Tarriño A (2001) El sílex en la Cuenca Vasco Cantábrica y el Pirineo Navarro: caracterización y su aprovechamiento en la Prehistoria. PhD Thesis (Universidad del País Vasco, Vitoria-Gasteiz).
14. Rios-Garaizar J. El nivel IXb de Ekain (Deba, Gipuzkoa): Una ocupación efímera del Auriñaciense Antiguo. Munibe (Antropologia-Arkeologia) 2011;62: 87–100.
15. Bon F. L’Aurignacien entre mer et océan. Réflexion sur l’unité des phases anciennes de l’Aurignacien dans le Sud de la France. Paris, Société préhistorique française, mémoire 2002;XXIX: 13–253.
16. Féblot-Augustins J. La circulation des matières premières au Paléolithique. Synthèse des données, perspectives comportementales. Études et Recherches Archéologiques de l´Université de Liège 75. Liège: Université de Liège; 1997.
17. Dini M, Baills H, Conforti J, Tozzi C. Le Protoaurignacien de la Grotte La Fabbrica (Grosseto, Italie) dans le contexte de l’arc nord méditerranéen. Anthropologie 2012 ;116: 550–574.
18. Bazile F. Le premier Aurignacien en france méditerranéenne. Un bilan. Espacio, Tiempo y Forma, Serie I, Prehistoria y Arqueología 2002 ;15: 215–236.
19. Ortega I, et al. L’occupation de l’Aurignacien Ancien De Barbas III (Creysse, Dordogne). Résultats préliminaires sur la fonction du site. Paléo 2006 ;18: 115–142.
20. Slimak L, Pesesse D, Giraud Y. La grotte Mandrin et les premières occupations du Paléolithique supérieur en Occitanie orientale. Espacio, Tiempo y Forma, Serie I, Prehistoria y Arqueología 2002 ;15: 237–259.
21. Fernandes P, Morala A, Schmidt P, Séronie-Vivien MR, Turq A. Le silex du Bergeracois: état de la question. In: Bertran P, Lenoble A, editors. Quaternaire continental d'Aquitaine, Excursion AFEQ. Bordeaux: Université de Bordeaux; 2012. pp 22–33.
22. Michel A et al. Nouvelles fouilles sur le site aurignacien Chez les Rois (Mouthiers-sur-Boëme, Charente). In: Jaubert J, Bordes J-G, Ortega I, editors. Les sociétés du Paléolithique dans un grand Sud-Ouest de la France: nouveaux gisements, nouveaux résultats, nouvelles méthodes. Mémoires de la Société Préhistorique Française XLVII; 2008. pp 289–299.
23. Chiotti L, Leoz LE, Nespoulet R, Pottier C. Quelques exemples de stratégies d’approvisionnement dans l’Aurignacien et le Gravettien à l’abri Pataud (Dordogne). In: Les matières premières lithiques en Préhistoire. Cressensac: Association Préhistoire du Sud-Ouest ; 2003. pp 115–122.
24. Bordes J-G, Bon F, Le Brun-Ricalens F. Le transport des matières premières lithiques à l’Aurignacien ancien entre le Nord et Sud de l’Aquitaine: faits attendus, faits nouveaux. In: Jauber J, Barbaza M, editors. Territoires, déplacements, mobilité, echanges durant la préhistoire Paris: CTHS; 2005. pp 185–198.
25. Normand C, et al. Nouvelles données sur la séquence aurignacienne de la grotte d'Isturitz (commune d'Isturitz et de Saint-Martin-d'Arberoue; Pyrénées-Atlantiques). In: Evin J, editor. Un siècle de construction du discours scientifique en Préhistoire. Paris: Mémoires de la Société Préhistorique Française; 2007. pp 277–293.
26. Bordes JG. News from the West: a reevaluation of the classical Aurignacian sequence of the Périgord. In: Bar-Yosef O, Zilhão J, editors. Towards a definition of the Aurignacian. Lisboa: Trabalhos de Arqueologia 45; 2006. pp 147–171.
27. Ziesaire P. Der Aurignacien-Fundplatz Altwies-Laangen Aker in Luxemburg. Luxembourg: Société Préhistorique Luxembourgeoise; 1998.
28. Miller R. Lithic Resource Management during the Belgian Early Upper Palaeolithic: Effects of Variable Raw Material Context on Lithic Economy. Liège: Eraul 91; 2001.
29. Šída P, Vokounová Franzeová D, Moravcová M. Proměny využívání zdrojů kamenných surovin v mladém a pozdním paleolitu a mezolitu Čech. Živá Archaeologie 2014;16: 17–24
30. Burkert W, Floss H. Lithic Exploitation Areas in the Upper Palaeolithic of West and Southwest Germany. A Comparative Study. In: Weisgerber G, Körlin G, editors. Stone Age – Mining Age. Bochum: Veröffentlichungen aus dem Deutschen Bergbau-Museum Bochum; 2005. pp 35–49.
31. Steguweit L. Retuschierte Lamellen im Inventar der Aurignacien-Station Alberndorf (N.-Ö.). Acta Archaeologica Carpathica 2009;XLII-XLIII: 7–25.
32. Moreau L, et al. Terrain difficulty as a relevant proxy for objectifying mobility patterns and economic behaviour in the Aurignacian of the Middle Danube region: the case of Stratzing (Austria). In: Pereira T, Terradas X, Bicho N, editors. The Exploitation of Raw Materials in Prehistory: Sourcing, Processing and Distribution. Newcastle: Cambridge Scholars Publishing; 2017. pp 134–147.
33. Bietti A, et al. Inorganic raw materials economy and provenance of chipped industry in some stone age sites of northern and central Italy. Coll Antropol 2004;28: 41–54.
34. Mussi M, Gioia P, Negrino F. Ten small sites: the diversity of the Italian Aurignacian. In: Bar-Yosef O, Zilhão J, editors. Towards a Definition of the Aurignacian. Lisboa: American School of Prehistoric Research/Instituto Português de Arqueologia; 2006. pp 189–209.
35. Bertola S, et al. La diffusione del primo Aurignaziano a sud dell'arco alpino. Preistoria Alpina 2013; 47: 17–30.
